# A Comparative Analysis of Single Versus Multiple Arterial Grafts in Coronary Arterial Bypass Grafting: Initial Experience in Iraq

**DOI:** 10.7759/cureus.66009

**Published:** 2024-08-02

**Authors:** Shkar R Haji Saeed

**Affiliations:** 1 Branch of Clinical Sciences, College of Medicine, University of Sulaimani, Sulaimani, IRQ; 2 Department of Cardiac Surgery, Sulaimani Cardiac Hospital, Sulaimani, IRQ

**Keywords:** coronary artery bypass grafting (cabg), mortality, cross-clamp duration, bypass duration, stroke, myocardial infarction, single arterial grafting, multiple arterial grafting

## Abstract

Introduction: The global recognition of multiple arterial grafting (MAG) and total arterial grafting (TAG) in coronary artery bypass grafting (CABG) is increasing. However, many centers have not yet adopted these procedures. Our study aims to examine the intraoperative, early postoperative, and two-year follow-up outcomes associated with MAG and TAG in candidates for CABG. The goal is to provide valuable insights into the role of these procedures.

Methods: A prospective comparative study was conducted at Sulaimani Cardiac Hospital to analyze a cohort of 300 patients who underwent CABG surgery between January 2021 and April 2022. Convenience sampling was used to select participants. Prior to surgery, patients underwent comprehensive pre-operative evaluations, with certain CABG types being excluded. The patients were then categorized into three groups based on their surgical approach: single arterial conduit (SA), two arterial conduits (MA), and total arterial revascularization (TA). The standard bypass procedure was performed for all patients, and they were monitored for 30 days, six months, and two years after the surgery. A range of variables, including bypass and cross-clamp times, as well as postoperative complications such as bleeding and stroke, were recorded and analyzed. Statistical Product and Service Solutions (SPSS, version 25; IBM Corp., Armonk, NY) was used for this analysis, with a predetermined significance threshold of p ≤ 0.05.

Results: The study included 300 participants who underwent CABG. The participants had an average age of 61.19 ± 4.67 years (95% CI: 36-81) and an average BMI of 27.40 ± 8.4 kg/m² (95% CI: 18-45). Diabetes was present in 40.3% of the patients, and the majority of the participants were male (77.7%). The number of vessels involved in the bypass varied, with two vessels in 21% of cases, three vessels in 65%, and four vessels in 14%. The left internal thoracic artery (LITA) was primarily used for arterial revascularization, and additional arterial conduits were used in 30.3% of cases. Statistical analysis showed significant differences in the number of grafts among patient groups (P = 0.042). However, there were no significant differences in bypass duration, cross-clamp duration, stroke incidence, or in-hospital mortality rates among the groups (P > 0.05). The rates of myocardial infarction (MI) approached significance (P = 0.05), and the mortality rates were comparable over a two-year period after CABG (4.7%) and at shorter intervals. These findings highlight the importance of age and the number of grafts in determining outcomes in CABG patients.

Conclusion: In a developing nation, the lack of a specialized center for MAG does not hinder the implementation of MAG or TAG. The overall occurrence of complications after CABG is similar across all groups, except for MI. Patients who undergo MAG have higher rates of overall survival compared to those who receive SA and TAG.

## Introduction

Ischemic heart disease (IHD) remains the leading cause of global mortality, highlighting the ongoing need for advancements in coronary revascularization strategies. The introduction of the cardiopulmonary bypass (CPB) machine in the 1950s was a significant milestone in cardiac surgery, enabling safer and more extensive CABG procedures. By the early 1990s, the use of the left internal thoracic artery (LITA) for the left anterior descending artery (LAD) had become the preferred method due to its higher rates of graft patency and better clinical outcomes [1]. Despite this, the adoption of the radial artery (RA) as a secondary graft has been relatively slow, with recent data indicating its utilization in less than 10% of cases across many United States (US) centers, despite its potential advantages in certain patient groups [2].

In recent decades, there has been a shift towards the use of MAG and TAG in CABG surgery, aiming to improve long-term outcomes by mimicking the natural integrity of the coronary circulation [3,4]. Despite the theoretical benefits, the optimal implementation of MAG and TAG varies among surgical centers worldwide, reflecting the complex interplay of patient-specific factors such as age, comorbidities, and the complexity of coronary lesions [5,6]. Additionally, technical considerations, including the surgeon's expertise in harvesting and anastomosis of arterial conduits, play a crucial role in the success of these advanced grafting techniques [5,6].

Although emerging evidence suggests potential long-term advantages of MAG and TAG over traditional single grafting methods, comprehensive data on perioperative and short-term outcomes are limited [2,4,7]. In this context, the concept of a specialized center for arterial grafting, characterized by a high utilization rate of second arterial conduits in CABG, raises important questions about its impact on procedural safety, effectiveness, and overall clinical outcomes [4]. To address these knowledge gaps, our study aims to examine the intraoperative, early postoperative, and two-year follow-up outcomes associated with MAG and TAG in candidates for CABG, with the goal of providing valuable insights into the role of specialized surgical expertise in optimizing coronary revascularization strategies.

## Materials and methods

Study design and patient selection

A prospective comparative study was conducted at a single center involving 300 patients who underwent CABG between January 2021 and April 2022. Data collection was done using a convenience sampling method. Prior to the surgery, all patients underwent coronary angiography and were referred to Sulaimani Cardiac Hospital in Sulaimani, Iraq. Patient preparation included transthoracic echocardiography to rule out valvular diseases and congenital anomalies. Demographic data such as age, gender, BMI, and diabetes status were recorded for each patient. The study included patients undergoing solitary CABG, elective CABG, and transsternal CABG. Patients who underwent off-pump CABG, urgent CABG, concomitant cardiac surgeries, and minimally invasive CABG procedures were excluded from the study. Patients were divided into three groups based on the type of conduits used for revascularization. Group SA consisted of patients who received a single arterial conduit, mainly the LITA. Group MA included patients who received a second arterial conduit using both LITA and the RA. Group TA comprised patients who underwent total arterial revascularization involving LITA and RA, with or without the right internal thoracic artery (RITA). In cases where a second arterial graft was used, the targeted vessel stenosis was more than 70%. All patients were admitted on the morning of the surgery and underwent sternotomy for CABG. Each patient was put on cardiopulmonary bypass. The LITA was harvested using the pedicled method before bypass initiation. The RA was harvested in an open, skeletonized manner from the non-dominant hand, after excluding peripheral vasculopathy and performing the Allen test. The RITA was also harvested using the pedicled method before bypass. Postoperative monitoring for 30 days included tracking variables, such as bypass time, cross-clamp time, the number of coronary arteries bypassed, and the use of arterial conduits. The observations extended to the length of hospital stay and early postoperative complications such as bleeding requiring re-exploration, stroke, and mortality. Follow-up assessments at six months and two years evaluated morbidity and mortality rates.

Ethical approval and informed consent

This study was approved by the College of Medicine, University of Sulaimani, with the ID number 301/17 on January 14, 2021. Informed consent was obtained from all participants. The study strictly adhered to the ethical principles outlined in the Declaration of Helsinki, which emphasize the importance of respecting individuals, their right to make informed decisions, and the need to ensure their well-being and confidentiality. All procedures involving human participants were conducted in accordance with these ethical standards.

Data analysis

The dataset was evaluated using appropriate statistical validation techniques to determine if it followed a normal distribution. Descriptive statistics were used to summarize the dataset, including measures such as the mean, standard deviation, and frequency and percentage distributions. To facilitate comparison across multiple groups, the study used a one-way analysis of variance (ANOVA) and a chi-square test. Survival analysis was conducted using the Kaplan-Meier method, which was particularly important for assessing the impact of interventions on different survival outcomes. All statistical analyses were performed with a significance threshold of p ≤ 0.05, ensuring a comprehensive and rigorous assessment and interpretation of the study's findings. The statistical software packages Statistical Product and Service Solutions (SPSS, version 25; IBM Corp., Armonk, NY) were used for data analysis.

## Results

The study included a cohort of 300 participants who underwent CABG. The participants had an average age of 61.19 ± 4.67 years (95% CI: 36-81) and an average BMI of 27.40 ± 8.4 kg/m^2^ (95% CI: 18-45). Among all the patients, 121 (40.3%) had diabetes. The majority of the patients, 233 (77.7%), were male. In terms of the number of vessels requiring bypass, the results showed that 63 (21%), 195 (65%), and 42 (14%) required bypass for two, three, and four coronaries, respectively. In relation to arterial revascularization, out of the 300 patients, 209 (69.7%) received one artery, with LITA being used in all patients, and the remaining conduits were veins. In addition, 53 (17.7%) received a second arterial conduit, with the RA being the second choice after LITA, and 38 (12.7%) received total arterial revascularization. This indicates that 30.3% of all patients received a second arterial graft or more (Table 1).

**Table 1:** The demographic features of all the patients. BMI = body mass index; SA = single artery plus vein; MA = second artery plus vein; TA = total arterial; SD = standard deviation; CI 95% = confidence interval. The data represented as frequency, percentage, mean, and standard deviation.

Variables	Frequency	Percentage
Gender		
Male	233	77.7%
Female	67	22.3%
Number of Affected Vessels		
Two	65	21.6%
Three	195	65%
Four	40	13.4%
Type of Grafts		
SA	209	69.67%
MA	53	17.67%
TA	38	12.7%
Presence of Diabetes	121	40%
Age	(Mean ± SD)	CI 95%
	61.19 ± 4.67	36-81
BMI (kg/m^2^)	27.40 ± 8.4	18-45

The age distribution results among the three groups demonstrated a statistically significant difference. The mean ages for groups SA, MA, and TA were 63.71, 55.77, and 54.87, respectively, with a p-value of 0.000. In contrast, the mean BMI values for the Groups were 27.42, 27.03, and 27.82, with a p-value of 0.73. The gender distribution, however, did not exhibit a significant difference (P = 0.78). Group SA consisted of 155 (74.16%) males and 54 (25.84%) females, Group MA had 45 (84.90%) males and eight (15.10%) females, and Group TA had 33 (86.84%) males and five (13.16%) females. The prevalence of diabetes was also similar (P = 0.243), with Group SA having 88 (42.11%) diabetics, Group MA with 16 (30.19%), and Group TA with 17 (44.74%). The number of grafts varied significantly (P = 0.042). In Group SA, there were 46 (22%) patients with two grafts, 133 (63.64%) with three grafts, and 30 (14.36%) with four grafts. In Group MA, there were five (9.43%) patients with two grafts, 39 (73.59%) with three grafts, and nine (16.98%) with four grafts. In Group TA, there were 14 (36.84%) patients with two grafts, 23 (60.53%) with three grafts, and one (2.63%) with four grafts. Bypass times were not significantly different, averaging 106.38 minutes for Group SA, 107.70 minutes for Group MA, and 100.50 minutes for Group TA (P = 0.45). Similarly, cross-clamp times showed no significant difference, with means of 62.84, 63.66, and 64.68 minutes, respectively (P = 0.8). Stroke incidence was uniformly high across groups, with no significant variation (P = 0.73). Myocardial infarction occurrence was marginally significant (P = 0.05), observed in one patient in Group SA, one in Group MA, and two in Group TA. Re-exploration rates did not differ significantly (P = 0.12), with 10 cases in Group SA, five in Group MA, and none in Group TA. In-hospital mortality rates were similarly non-significant (P = 0.51), with five deaths in Group SA, none in Group MA, and one in Group TA. These findings emphasize that age and graft number are the main variables that display significant differences among the patient groups (Table 2).

**Table 2:** The differences among the three groups (SA, MA, and TA). BMI = body mass index; MI = myocardial Infarction; SA = single artery plus vein; MA = second artery plus vein; TA = total arterial; Mins = minutes; P ≤ 0.05. The ANOVA and chi-square tests were used. The data represented as frequency, percentage, and minutes.

Variables	Group SA (N =209)	Group MA (N=53)	Group TA (N=38)	P value
Age (mean)	63.71	55.77	54.87	0.000
BMI (mean)	27.42	27.03	27.82	0.73
Gender N (%)				0.78
Male	155 (74.16%)	45 (84.90%)	33 (86.84%)	
Female	54 (25.84%)	8 (15.10%)	5 (13.16%)	
Diabetes N (%)				0.243
Yes	88 (42.11%)	16 (30.19%)	17 (44.74%)	
No	121 (57.89%)	37 (69.81%)	21 (55.26%)	
Number of grafts N (%)				0.042
Two	46 (22%)	5 (9.43%)	14 (36.84%)	
Three	133 (63.64%)	39 (73.59%)	23 (60.53%)	
Four	30 (14.36%)	9 (16.98%)	1 (2.63%)	
Bypass time (minutes)	106.38	107.70	100.50	0.45
Cross clamp time (minutes)	62.84	63.66	64.68	0.8
Stroke N (%)				
Yes	203 (97.13%)	51 (96.23%)	36 (94.74%)	0.73
No	6 (2.87%)	2 (3.77%)	2 (5.26%)	
MI (n)	1 (0.48%)	1 (1.88%)	2 (5.26%)	0.05
Re-exploration N (%)	10 (4.78%)	5 (9.43%)	0 (00%)	0.12
Death in hospital N (%)	5 (2.39%)	0 (00%)	1 (2.63%)	0.51

**Figure 1:**
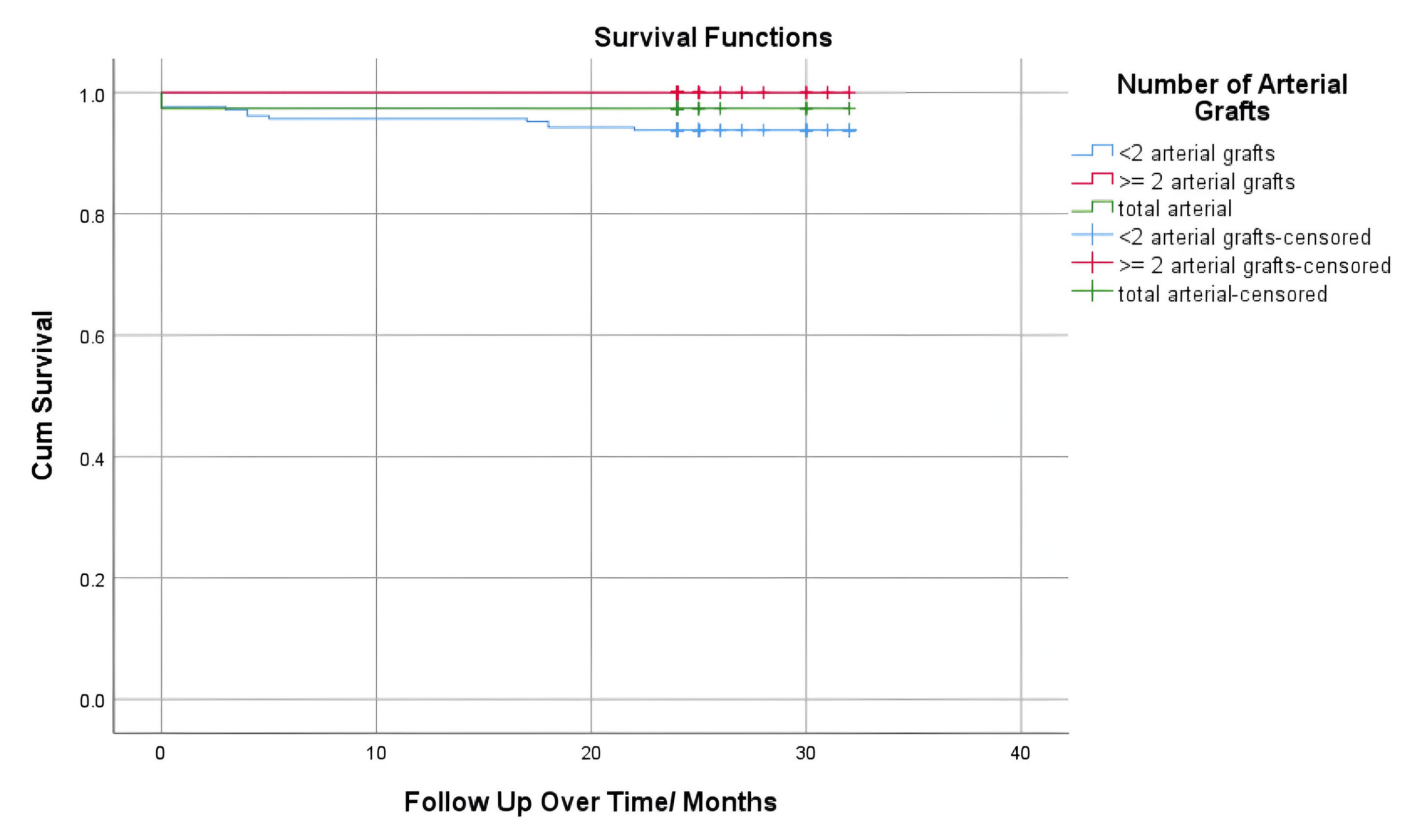
Kaplan-Meier graph showing the survival analysis between groups. The data represented as months.

In relation to the survival of the mentioned groups, our study found that six deaths occurred within the first month. Out of these, four patients survived the first 30 days but later passed away within the following six months. Additionally, four more patients died within the first two years after surpassing the initial six-month period. Therefore, a total of 14 individuals (4.7%) passed away within the two-year post-CABG period. When comparing the mortality rates at different time points - one month, six months, and two years - no statistically significant differences were observed among the groups. The average follow-up period for the groups was 23.97 months (Figure 1).

## Discussion

The results of our study indicate that, initially, there was no difference in the duration of bypass time and cross-clamp time between the groups. Although the difference is not statistically significant, the MA group displayed higher survival rates compared to both the SA and TA groups. All groups had similar rates of major post-surgical complications, except for a slightly higher occurrence of MI in the total arterial group. To be precise, there were two cases (5.26%). Furthermore, we discovered a correlation between post-surgery stroke and higher mortality rates. In the context of patients undergoing CABG, it is well-established that age is an independent factor that influences the risk and outcome of the surgery [1-3]. It is widely recognized that younger individuals with fewer medical conditions are more likely to receive multiple arterial grafts [7]. In our study, Group MA and Group TA had mean ages of 55.7 and 54.8 years, respectively, while Group SA had a mean age of 63.7 years.

The impact of cross-clamp time during surgery on mortality and complications has been extensively studied [8,9]. Al-Sarraff et al. found that each minute beyond 60 minutes of cross-clamp time increased overall mortality by 2% across all risk categories [8]. Another study demonstrated that cross-clamp times exceeding 75 minutes were associated with higher mortality rates [8]. Additionally, a study showed that total arterial grafting requires longer cross-clamp time due to the technical expertise needed for handling arterial conduits compared to venous conduits [10]. In our research, we observed no significant difference in cross-clamp times among the groups (62.84 vs 63.66 vs 64.68 minutes) with a P value of 0.81. However, a noteworthy finding from our study was that patients who received more than one artery graft had zero mortality during the two-year follow-up period. This improved survival rate in the multiple arterial graft group compared to the total arterial graft group presents conflicting outcomes. The better survival rate in the multiple arterial graft group compared to the single arterial graft group might be attributed to the age difference (55.7 vs 63.7). Nonetheless, the age difference was not substantial when compared to the total arterial graft group.

Existing studies generally support the idea that multiple arterial grafting improves survival rates [1,2,7,10-16]. For example, Rosenblum et al. demonstrated a survival advantage in midterm follow-up for multiple arterial revascularizations compared to single arterial revascularization, regardless of completeness [7]. However, no significant difference was observed between the two in short-term follow-up [7]. On the contrary, Rocha et al. found in their 8-year follow-up study that total arterial grafting did not show any superiority over multiple arterial grafting in terms of in-hospital mortality and late complications [11]. In our patient population, we opted to use the radial artery as the secondary conduit based on supporting studies [2,3]. The radial artery had better outcomes compared to the right internal thoracic artery (RITA) and the saphenous graft [1,2]. Interestingly, while only a small percentage of patients in most US and EU centers receive a second arterial graft [17], we offered this option to a larger proportion of our patients. A meta-analysis by Samadashvili et al. found that the volume of multiple arterial grafting performed by specialized centers did not always lead to better long-term survival outcomes [4]. This suggests that specialized centers are not necessary for successful multiple arterial grafting procedures [8]. The literature does not show a significant difference in major complications, such as stroke and MI, between single arterial and multiple arterial grafting techniques [7]. Our study had similar results, except for a higher incidence of MI in the total arterial group.

This study has several limitations worth noting. Firstly, the research was conducted at a single medical center, which may limit the applicability of the findings to other institutions or geographic regions. Additionally, the sample sizes in Groups MA (n=53) and TA (n=38) were relatively small, potentially impacting the ability to detect subtle yet clinically significant differences among the groups. The lack of long-term follow-up data also restricts the assessment of the lasting effects of single versus multiple arterial grafts on patient survival and postoperative quality of life. Moreover, the study did not account for various potentially influential factors, such as patient comorbidities, variations in postoperative care, and differences in surgeon experience, all of which could affect the outcomes. Consequently, these limitations highlight the need for further research, ideally through multi-center studies with larger sample sizes and longer follow-up periods, to validate and expand upon these initial findings.

## Conclusions

The use of multiple arterial grafts in patients undergoing CABG has become popular worldwide. This two-year follow-up study on elective CABG patients has shown that incorporating multiple arterial grafts improves patient survival rates and reduces morbidity without significantly increasing the duration of the surgical procedure or cross-clamp time. Although the statistical significance of the difference in survival rates was not notable, Group MA consistently produces better outcomes compared to single Group SA and Group TA. It is important to emphasize that the decision to perform multiple arterial grafting should not depend solely on the availability of a specialized center for this procedure. In certain cases, the radial artery may offer a more beneficial alternative to the greater saphenous vein for specific patients. This finding underscores the clinical importance of multiple arterial grafts in enhancing patient outcomes, despite the lack of statistical significance.
